# Bi-layered Nanofibers Membrane Loaded with Titanium Oxide and Tetracycline as Controlled Drug Delivery System for Wound Dressing Applications

**DOI:** 10.3390/polym11101602

**Published:** 2019-10-01

**Authors:** Abdelrahman I. Rezk, Ji Yeon Lee, Byeong Cheol Son, Chan Hee Park, Cheol Sang Kim

**Affiliations:** 1 Department of Bionanosystem Engineering, Jeonbuk National University, Jeonju, Jeonbuk 561-756, Korea; abdelrahmanrezk58@gmail.com (A.I.R.); Rezk@jbnu.ac.kr (B.C.S.); 2 Division of Mechanical Design Engineering, Jeonbuk National University, Jeonju, Jeonbuk 561-756, Korea; swc2736630@gmail.com

**Keywords:** composite nanofibers, electrospinning, tetracycline, local drug delivery, wound dressing

## Abstract

The aim of this study is to develop a novel functional bi-layered membrane loaded titanium oxide (TiO_2_) and tetracycline (TTC) for application in wound dressing. The advantages of the electrospinning technique have to be considered for the uniform distribution of nanoparticles and TTC drug. The as prepared nanofibers and TiO_2_ were characterized in terms of morphology, fiber diameter, mechanical properties and surface wettability. The in vitro drug release study revealed initial burst release followed by a sustained control release of TTC for 4 days. The in vitro antibacterial of the bi-layered nanofibers was conducted against Gram-positive (*Staphylococcus aureus*) and Gram-negative (*Escherichia coli*) bacteria species showing excellent antibacterial effect for drug loaded samples compared with PCL nanofibers. Subsequently, cell counting kit-8 (CCK-8) and confocal laser scanning microscopy (CLSM) were used to evaluate its biocompatibility in vitro. Our results revealed that the bi-layered membrane has better antibacterial and cell compatibility than the control fiber. This suggests that the fabricated biocompatible scaffold is appropriate for a variety of wound dressing applications.

## 1. Introduction

Wound dressings are attracting substantial, widespread attention in chronic wound therapy, such as for diabetic chronic ulcers, which remains a significant clinical challenge due to the limited treatment routes and suboptimal healing outcomes. Therefore, developing an effective wound dressing treatment would be greatly beneficial [1,2].

An ideal wound dressing scaffold should have the same function and structurally similar to skin tissue in order to promote cell proliferation and adhesion, isolate the wound from adverse environments and avoid secondary damage to the wound, thus providing mechanical strength and accelerating the wound healing [2]. In order to mimic the assembly and function of skin, wound dressings with a bi-layered model have been introduced. Examples include Biobrane™ (silicone, nylon mesh, and collagen) and OASIS (porcine acellular lyophilized small intestine mucosa) [3]. The scaffolds provide a barrier against bacterial infection and water loss, and also have a tridimensional structure with interconnected pores in wounds. However, these scaffolds still have some disadvantages, including cost-associated concerns, infection risks factors, and temporary coverage [4,5].

Incorporation of nanotechnology into the biomaterials has recently been gaining a lot of interest as it is considered the second generation in the nanotechnology world. It refers to the assemblies of hetero- or homo-nanomaterials structures to the biomaterials for different purposes. The nanocomposites play an important role in this nanotechnology, which can enhance their properties and reveals new functionalities of using metal oxide nanomaterials with bio polymers [6,7].

Several biopolymers scaffolds have been developed as one of the main components for biomedical application; for example, tissue engineering and novel biomedical technologies such as regenerative medicine and controlled drug delivery [8]. The most commonly used biomaterials in medical applications are polymers including polylactides, chitosan, polyvinyl alcohol, polyglycolide, polydioxanone and polycaprolactone (PCL) [9]. PCL is Food and Drug Administration (FDA) approved as a semi-crystalline polymer, possessing many advantages such as its use in implantable devices, and is known for its excellent mechanical properties, biodegradation, biocompatibility, chemical stability and tissue-compatible nature. PCL was tested and approved to be used in drug delivery vehicles or tissue engineering scaffolds [10]. Polydioxanone (PDO) is derived from the paradioxanone monomer via a ring-opening polymerization with heat and catalyst-like zirconium acetylacetone, diethylzinc [11,12]. PDO is a crystalline, colorless, bioabsorbable polymer, which was developed specifically for wound dressing sutures. Ethicon Inc., A Johnson and Johnson Company, markets it in the United States. Furthermore, PDO loses half of its strength after four weeks of in vivo implantation. Compared to other bioabsorbable polymers such as polyglycolic acid and polylactic acid, PDO is emerging as an attractive bioabsorbable polymer due to its excellent biodegradability without tissue inflammation [10,13,14].

Metal oxide nanomaterials show outstanding property in signaling the tissue regeneration, biocompatibility, antibacterial effect, in wound dressing applications [15,16]. Titanium dioxide (TiO_2_) is a white solid inorganic material and the ninth most common element in the world [17]. Nanoparticles size TiO_2_ play a crucial role to enhance the mechanical properties, antibacterial effectiveness against both Gram-positive and Gram-negative bacteria, biocompatiblity and high corrosion resistance [18]. TiO_2_ nanotube materials have been widely studied as adhesion and growth support platforms for tissue regeneration, inhibition of bacterial adhesion and drug delivery [19,20]. Previous study developed bilayer composite and film based on TiO_2_ nanostructured for wound healing applications [21]. Their study revealed that the addition of TiO_2_ nanostructured into chitosan and pectin increased tensile strength, excellent antimicrobial activity, decent biocompatibility and faster wound healing.

We designed a bi-layered composite nanofibers composed of PDO-incorporated tetracycline drug as the inner layer with an antimicrobial function against Gram positive and Gram negative bacteria, as well as a PCL fundamental layer to maintain the mechanical properties and protect from external moisture due to its low wettability and hydrophobic nature. In particular, PCL nanofibrous membranes fabricated by electrospinning are attracting a lot of interest for various applications, because they have numerous useful properties including high surface area, biodegradability, biocompatibility, and excellent mechanical properties [22]. PDO composite nanofibrous mat-loaded antibacterial agents, such as Ag nanoparticles, ciprofloxacin and tetracycline, are attractive wound dressing materials due to their excellent antibacterial activity against Gram-positive and Gram-negative bacteria [23,24,25]. Tetracycline (TTC) is one of the most commonly antibiotics for the treatment of several skin infections [26], such as acne, periodontal, and urinary infections. The antibacterial action of TTC was tested for different microorganism agents and showed low minimal inhibition concentration. In this study, we investigated the use of a novel bilayer composite for full-thickness wound healing. We expected that the PPTT (PCL–PDO–TiO_2_–TTC) composite nanofiber would help to protect the wound area from bacterial infection while the ECM sheet would promote fibroblast cell regeneration.

## 2. Materials and Methods

### 2.1. Materials 

In this study, poly (ɛ-caprolactone) (PCL, *M*_w_ = 70,000–90,000, Sigma Aldrich Chemical Co. Ltd. (Seoul, Korea), Polydioxanone (PDO) (Resomer^®^ X 206 S, Sigma–Aldrich, USA), and 1,1,1,3,3,3-hexafluoroisopropanol (HFIP) were purchased from Tokyo Chemical Industry Co., Ltd. (Tokyo, Japan). TiO_2_ (Sigma Aldrich, 25 nm particle size) Tetracycline hydrochloride ≥95% (European Pharmacopoeia HPLC assay, Sigma–Aldrich, (St. Louis, MO, USA). Chloroform (99.5%, Samchun, Pyeongtaek, Korea), 1,1,1,3,3,3-Hexafluoro-2-propanol (HFIP), Sigma–Aldrich Chemical Co. Ltd. (Seoul, Korea) and Methyl alcohol (Samchun chemical, Pyeongtaek, Korea).

### 2.2. Solution Preparation for Electrospinning

The dual layers of electrospun nanofibers were produced via the electrospinning setup described here. In this study, the PCL layer was fabricated by using 12 wt% of PCL solution (chloroform-methanol, 3:1). The second layer of nanofiber was PDO loaded with TiO_2_ nanoparticles. Here, 8 wt% PDO was dissolved in HFIP and mixed with 3, 5 wt% TiO_2_, and 5 wt% tetracycline of the total polymer weight, and the mixture composite was ultrasonicated for 15 min prior to electrospinning. The electrospinning process progressed at a flow rate of 1 ml/h using a high DC voltage of 17 KV for the PCL layer and 20 KV for the second layer using needle gauge 21; the distance was maintained at 15 cm and the electrospun nanofibers were collected on the rotating drum using a polyethylene sheet, then dried in a vacuum oven. Dual layer samples were referred to as PP3T5T (PCL–PDO–3%TiO_2_–5%TTC) and PP5T5T (PCL–PDO–5%TiO_2_–5%TTC).

### 2.3. Characterizations

The morphology for different samples of nanofibers and TiO_2_ nanoparticles were analyzed using field-emission scanning electron microscopy (FE-SEM, Carl Zeiss supra-40 VP, Germany). Image J (NIH, USA) software was used to determine the average fiber diameter for which 100 nanofibers were selected from the different images of the same sample to take the mean value. A transmission electron microscopy BIO-TEM (HITACHI H-7650, Japan) was used to analyze the morphology of TiO_2_ nanoparticles. The contact angle (wettability) was measured using the deionized water contact angle measurement system with a contact angle meter (Digidrop, GBX, France). Deionized water was automatically dropped onto the nanofiber sample and measured at 1, 3 and 5 s. The mechanical strength properties of different nanofibers samples were measured with a strain rate of 10.0 mm min^−1^ at room temperature by universal testing machine (MTDI Inc. Korea) with a 10 N KAF-TC load sensor, where each mat was prepared in standard shape (dog-bone shape). 

### 2.4. In Vitro Drug Release Study and Antibacterial Activity

The drug release from the bi-layered nanofiber mat was assessed by placing a known weight of nanofiber membrane into conical tubes containing PBS (10 mL, pH 7.4) and transferring the tubes into a shaking incubator previously set at 37 °C and 100 rpm (SI-300R, Lab companion). Then, at different determined time intervals, 3 mL of PBS release media was taken for sampling and then put back into the conical tubes [27]. Using a UV spectrophotometer, the amount of drug release was measured at a wavelength of 364 nm of maximum absorbance of tetracycline in PBS. The amount of tetracycline was determined by using *a* calibration curve constructed from known tetracycline concentration. Further, the calibration curve satisfies Lambert and Beer’s law:

y=ac+b

where A is absorbance, a is slope, b is intercept, and c designates the drug concentration.

Next, the dissolve tetracycline at various time intervals were plotted as the percentage of release versus time. In this test, samples were triplicated and average values were reported.

The antibacterial activity of Gram-positive *Staphylococcus aureus* (*S. aureus*) and Gram-negative bacteria *Escherichia coli* (*E. coli*) were investigated against PCL, PP3T5T, and PP5T5T by inhibition zone testing [28]. First, an agar plate was prepared (recipe: 1 g tryptone, 0.5 g yeast, 1 g NaCl, agar 1.5 g and water 100 mL, which is poured into each 8 mL of Petri dish and kept to solidity). Then, 0.5 mL of bacterial solution diluted to 10^6^ CFU/mL was added and incubated for 24 h. The nanofibrous mat samples were then placed on each agar plate containing bacteria. Finally, the agar plate was incubated at 37 °C for 24 h, and the diameters of the inhibition zone were recorded using Image J (NIH, USA) software.

### 2.5. In vitro Cell Culture Study

Fibroblast cells (NIH3T3E1) were cultured in D-Minimal Essential Medium (D-MEM, Hyclone) supplemented with 10% fetal bovine serum (FBS, GIBCO) and 1% penicillin-streptomycin in atmospheric condition (5% CO_2_ at 37 °C with 95% humidity). The different samples of nanofiber were cut into the circular shape, then fixed on cover slips that fit the 48-well plate and sterilized under ultraviolet (UV) radiation overnight, then washed in PBS. Then, fibroblast cells were seeded onto the different samples for 2, 4 and 6 days. The proliferation of cells on the substrate was investigated using Dulbecco’s cell counting kit-8 (CCK-8). NIH3T3E1 of 500 μL suspension (1 × 10^4^ cells/well) were left to attach onto the surface of different samples. The cell culture media was changed every 24 h. The proliferation of cells on the nanofiber samples was observed at 2, 4, and 6 days, where 50 μL of CCK-8 solution was added to each well followed by 2 h incubation at 37 °C, then 100 μL from each well was transferred to 96-well plates. The absorbance of each well plate content was evaluated at a wavelength of 450 nm using a sunrise microplate reader (Tecan, Austria).

In order to examine cell attachment and spreading, the NIH3T3E1 cells were cultured directly on the different samples at a seeding density of (20 × 10^3^ cells/well). The 2 and 4-day cells cultured samples were washed with PBS (pH 7.4) and fixed with 4% paraformaldehyde for 2 h, then washed with 20%, 30%, 50%, 75%, and 95% ethanol for 20 min. Next, they were dried overnight in a laminar flow hood. The cell morphology and adhesion were determined via FE-SEM. In addition to FE-SEM images, a confocal laser scanning microscope (CLSM) (Carl Zeiss, Japan) was employed to further confirm the cell biocompatibility, spreading behavior and penetration of the fibroblast cells on different samples. Accordingly, the cells were cultured on the scaffolds for a designated time (2 and 4 days); then, 4% paraformaldehyde was used to fix the cells for 30 min, the samples were washed with PBS solution, the cells were then permeabilized with triton and washed twice with PBS, then human serum albumin (HSA) was added to block the nonspecific binding site and the samples were washed twice with PBS before being stained with Rohadamine and DAPI (4′,6-diamidino-2-phenylindole) (Life Technologies) according to the manufacturer’s protocol. Finally, the stained nanofiber samples were transferred into a confocal dish that was mounted before being evaluated using confocal laser scanning microscopy.

## 3. Results and Discussion

A dual layer nanofibrous mat was successfully fabricated using an electrospinning method.

**Figure 1:**
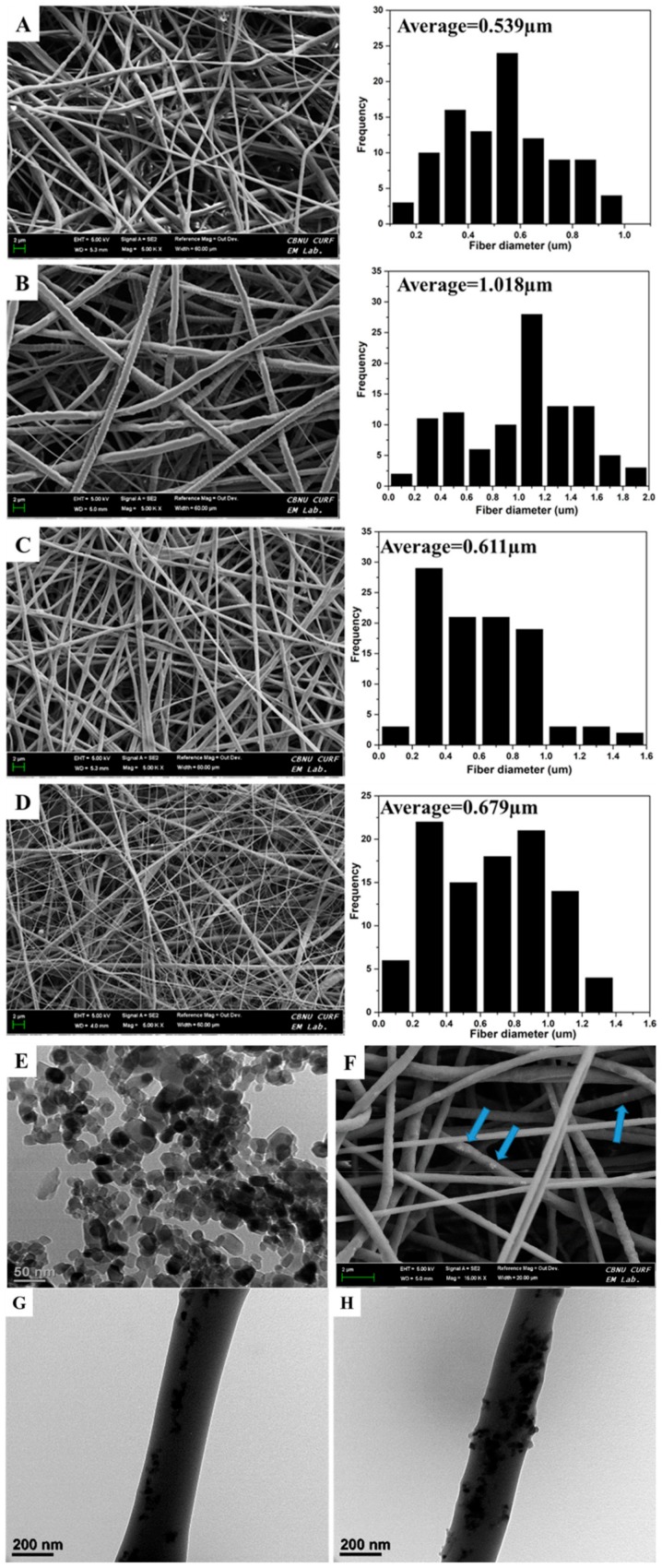
FESEM images and fiber diameter distribution of (**A**) PCL, (**B**) PDO, (**C**) PP3T5T, and (**D**) PP5T5T. (**E**) BioTEM image of TiO_2_ NPs and (**F**) FESEM image showing loaded particles in PP5T5T mat. (**G**) and (**H**) BioTEM images of PP3T5T and PP5T5T respectively. Dual layer samples were named PP3T5T (PCL–PDO–3%TiO_2_–5%TTC) and PP5T5T (PCL–PDO–5%TiO_2_–5%TTC).

Figure 1 shows the morphology of the different nanofibers produced of PCL, PDO, PP3T5T, and PP5T5T through selected FESEM images. As shown in Figure 1, there are bead free nanofibers with a smooth surface for each layer separately, and the dual layer mat displays the heterogeneous morphology of micro diameter nanofibrous and a thinner diameter of a web-like shape on the surface. The first layer represents pure PCL to maintain the mechanical properties of the nanofiber mat, while the second layered is composed of PDO loaded with nanoparticles and TTC drug molecules to provide an antibacterial effect. Figure 1A,B displays PCL and PDO nanofiber and Figure 1C,D displays the morphology of the bilayer nanofiber membrane after incorporating different concentration of TiO_2_ nanoparticles 3%, 5% and same drug content 5%. The bilayer membrane represented the uniform distribution of the nanofiber with a web-like shape, which was approximately similar to natural extracellular bone matrix with excellent biocompatibility and fibroblast cell attachment. 

Moreover, the average fiber diameter of pure PCL nanofiber was 0.539 µm and for second layer of PDO was 1.018 µm. while for PP3T5T and PP5T5T the average fiber diameter was 0.611 and 0.679 µm, respectively. Morphological alterations after adding TiO_2_ and TTC were observed and explained as the fiber diameter was decreased due the hydrogen bonding interaction between PDO and TiO_2_ which formed between hydroxyl and carbonyl group of the PDO, which in turn helps to increase their miscibility.

Bio-TEM images confirmed the presence of TiO_2_ nanoparticles inside the PDO nanofibers. Figure 1G,H displays Bio-Tem image of the PDO nanofiber loaded with 3% and 5% TiO_2_ nanoparticles respectively. From the TEM image, it is clear that the TiO_2_ nanoparticles were uniformly distributed inside the nanofibers. The nanoparticles loaded second layer of PDO enhanced the mechanical properties and the bio mineralization.

**Figure 2:**
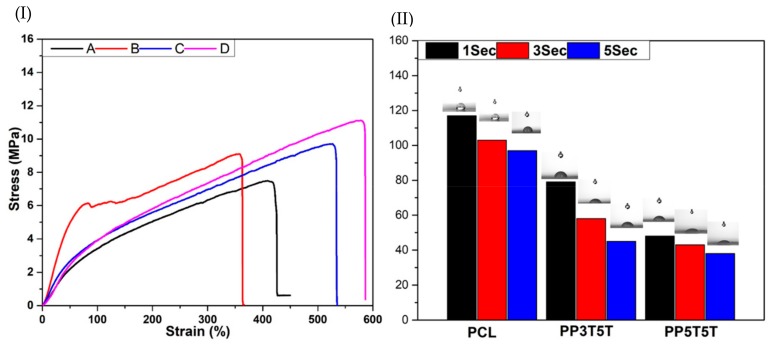
(**I**) Mechanical property of (**A**) PCL, (**B**) PDO, (**C**) PP3T5T, (**D**) PP5T5T. (**II**) Contact angle measurements of different samples after 1, 3 and 5 Sec.

Cell proliferation and tissue regeneration require sufficient mechanical support without new-tissue deformation. Therefore, bi-layered nanofibers were evaluated to confirm if they were suitable for regeneration application. Figure 2I shows the tensile strength curves for different samples. PP3T5T and PP5T5T bi-layered nanofibers presented excellent strength and elasticity compared to pure PCL or PDO nanofibers. We assumed that the addition of TiO_2_ nanoparticles increased the tensile strength of pure PCL or PDO samples, which could be explained by the intra and inter molecular hydrogen bonding between the TiO_2_, TTC and polymer molecules which caused the point bond structure [29]. From these results, we revealed that the mechanical strength of PP3T5T and PP5T5T nanofiber could fulfill the basic requirements for wound dressing application.

The wettability of the composite nanofibers mat is considered to be an essential property that affects the surface properties of the scaffold and hence affects drug release, the cells adhesion and proliferation. The contact angle of the bi-layered nanofiber mat PP5T5T was decreased dramatically compared to PCL, as shown in Figure 2II. Our results show that the outer layer of PCL displayed hydrophobic properties with a contact angle of 117° after 1 sec to protect the wound from the outer environment as the main function of the PCL layer; the second layer of the composite nanofiber PP5T5T and PP3T5T show lower contact angles of 79 and 48° after the same time duration, respectively, as shown in Figure 2II, as a result of adding nanoparticles and drug in way to enhance cell attachment and proliferation. The presence of TiO_2_ nanoparticles (polar surface groups) inside the PDO nanofiber enhanced its hydrophilicity due to a higher interaction between the composite mat and solvent [30,31].

**Figure 3:**
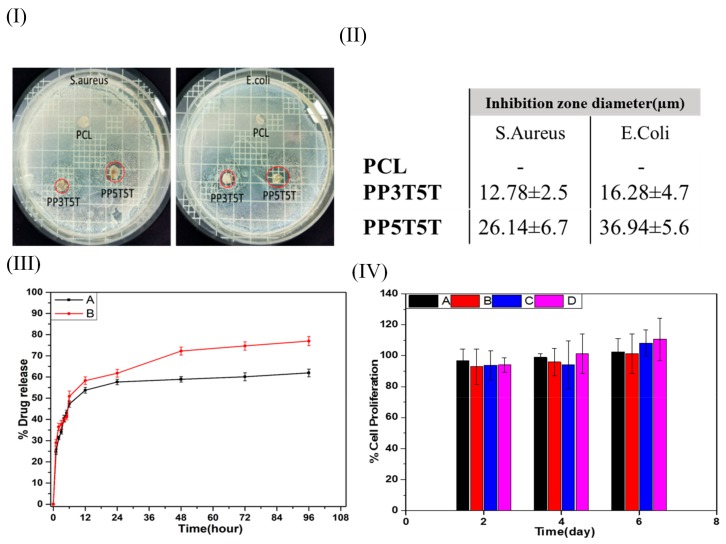
(**I**) Inhibition zone test and (**II**) table show inhibition zone diameter of different samples. (**III**) Drug release test from (**A**) PP3T5T and (**B**) PP5T5T. (**IV**) Cell biocompatibility of (**A**) PCL, (**B**) PDO, (**C**) PP3T5T, and (**D**) PP5T5T.

Clinical infections associated with surgical bio-implants are normally more challenging to control due to the required longer periods of antibiotic therapies and frequent surgical procedures. The antibacterial effects of pristine PCL, PP3T5T, and PP5T5T were studied quantitively and qualitatively using the inhibition zone method. Our findings revealed that PCL samples have no antibacterial effect while the drug loaded layer shows an antibacterial effect against both Gram-negative and Gram-positive bacteria species. As the nanoparticles, the concentration increased from 3% to 5% as the amount of the drug released was increased, which confirmed by the fact that the inhibition zone diameter was increased in PP5T5T samples, as shown in Figure 3I,II.

Figure 3III shows the drug release profile of TTC drug from different composite nanofiber (PP3T5T and PP5T5T) in the PBS solution for 4 days. The release profile of TTC from PP3T5T shows an initial burst release of 47.2% within the first 6 h, followed by slow release represented as a plateau within 4 days by releasing 61.9%. Further, the burst release of TTC drug molecules from PPT5T5 was 50.8% within the first 6 h, which was slightly higher than the release from PP3T5T. The drug release profile displayed a biphasic release profile, which can be explained in two steps; the initial diffusion from nanofibrous matrix and a second diffusion stage from the aqueous pores formed in the polymeric mat. For PP3T5T, the initial release of TTC from a nanofibrous mat was high due to the high amount of the drug near the nanofibrous surface. Indeed, TTC molecules tend to accumulate on the surface of the nanofibers during the electrospinning—this was previously explained as the low molecular weight and affinity to the aqueous solvent [32]. Then, release through diffusion in the second stage was extremely slow due to the low pore formation addition to the slow degradation of the polymer. By increasing nanoparticles concentration, the total TTC released was increased from 61.9% to 77% over 4 days. This was explained by the fact that the higher concentration of the nanoparticles results in faster solvation step, and as a result, the structure will be more open and easier for the drug to diffuse out. The results of this study show that the composite nanofiber can release TTC in a controlled manner, making it especially available as a controlled drug delivery system.

The cytotoxicity of the pure PCL, PDO, PP3T5T and PP5T5T composite nanofibers has been evaluated by means of CCK-8 assay. Figure 3IV shows the cell viability of fibroblast cells seeded on different composite nanofiber mats (PCL, PDO, PP3T5T and PP5T5T) after 2, 4, and 6 days of cell culturing. All of the samples show good biocompatibility without any cytotoxicity. Therefore, these results clearly showed that the TTC-loaded composite nanofibers are not toxic for cell activity and show excellent cell proliferation growth, which makes them applicable for wound dressing or tissue regeneration.

**Figure 4:**
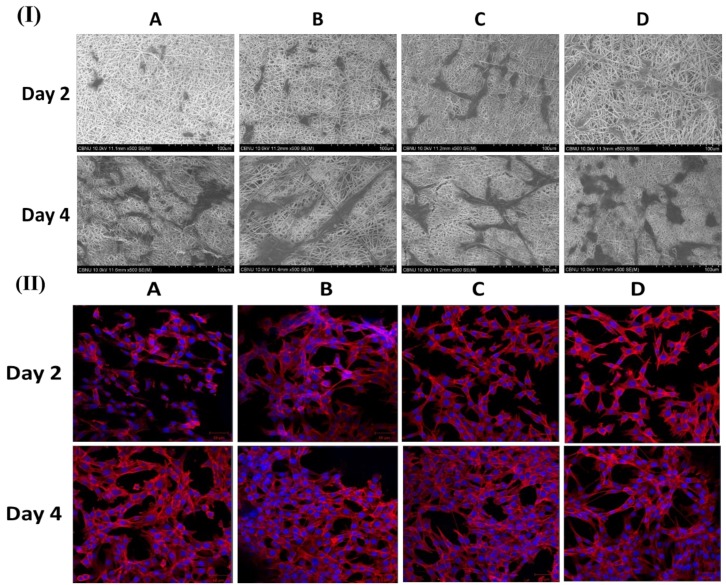
(**I**) FESEM and (**II**) CLSM images of Rhodamine/DAPI staining show fibroblast cells attachment on different samples of (**A**) PCL, (**B**) PDO, (**C**) PP3T5T, and (**D**) PP5T5T.

Figure 4I,II show FESEM and CLSM images of fibroblast cell attachment on different nanofibers mat (PCL, PDO, PP3T5T, and PP5T5T) after 2 and 4 days of cell culturing.

**Figure 5:**
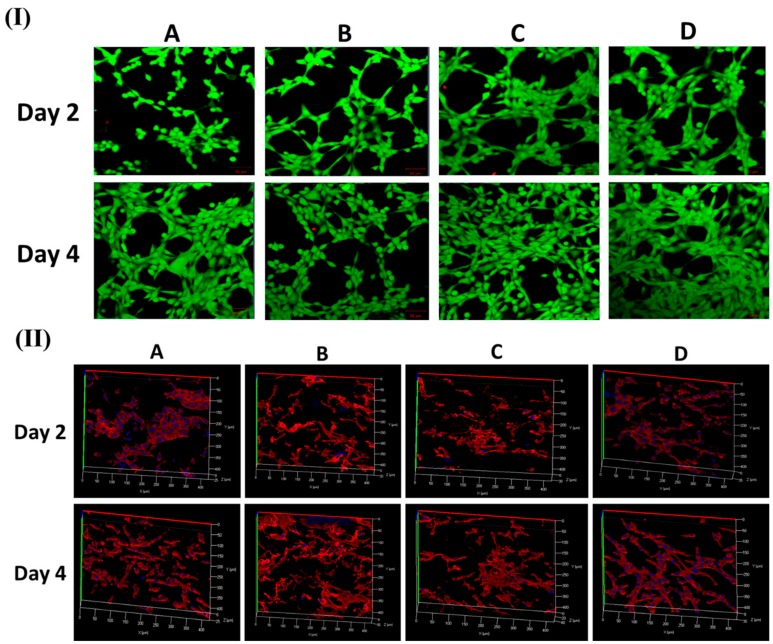
(**I**) Live dead assay of fibroblast cells on different mats. (**II**) Z-stack CLSM images of Rhodamine/DAPI staining show the penetration of fibroblast cells on different samples on different samples. (**A**) PCL, (**B**) PDO, (**C**) PP3T5T, and (**D**) PP5T5T.

The 2 day results show good cell adhesion and spreading of the cells on different samples, while after 4 days, the cells were fully extended and interconnected with each other. The presence of nanofiber mass beneath the osteoblast cells confirmed that fibroblast cells can penetrate and migrate within these mats in a manner similar to natural ECM [33]. Moreover, the morphology of the cells was further studied by CLSM images, as shown in Figure 4II confirming the excellent cell adhesion on the composite nanofibers. The CCK results show similar results for live and dead assay, as shown in Figure 5I. Figure 5II shows Z-stack data results that confirm the penetration of fibroblast cells through the nanofibers. The cells on the pure PCL and PDO are in fact not penetrated in the nanofibers as much as with the PP3T5T and PP5T5T. Presence of the cultured fibroblasts in the PCL was detected at a depth of 0 to 25 μm. The color images of fibroblasts on composite nanofibrous mats confirmed cellular penetration up to a depth of 40 and 35 μm for PP3T5T and PP5T5T, respectively, after 4 days of cell culture. Our composite nanofibrous mats permit greater cellular penetration, which is essential for wound healing.

## 4. Conclusions

In this study, a bi-layered membrane has been fabricated using the electrospinning technique. The outer layer of PCL has a hydrophobic nature to protect from the outer environment and retain its mechanical properties. The second layer loaded with tetracycline displayed a uniform distribution of the TTC drug along the nanofibers. Our results revealed that TTC-loaded nanofibers have a substantial antibacterial effect on *E. coli* and *S. aureus* sustained by a drug release. The bi-layered membrane shows fibroblast cell adhesion and proliferation superior to those of the control fibers, representing that the novel functional composite nanofiber mat could be used as a potential scaffold in applications such as controlled drug delivery and wound healing.
